# Thermostable and Long-Circulating Albumin-Conjugated *Arthrobacter globiformis* Urate Oxidase

**DOI:** 10.3390/pharmaceutics13081298

**Published:** 2021-08-19

**Authors:** Byungseop Yang, Inchan Kwon

**Affiliations:** School of Materials Science and Engineering, Gwangju Institute of Science and Technology (GIST), Gwangju 61005, Korea; yangbs@gist.ac.kr

**Keywords:** *Arthrobacter globiformis*, gout, half-life extension, inverse electron demand Diels-Alder reaction, site-specific albumin conjugation, thermostability, urate oxidase

## Abstract

Urate oxidase derived from *Aspergillus flavus* has been investigated as a treatment for tumor lysis syndrome, hyperuricemia, and gout. However, its long-term use is limited owing to potential immunogenicity, low thermostability, and short circulation time in vivo. Recently, urate oxidase isolated from *Arthrobacter globiformis* (AgUox) has been reported to be thermostable and less immunogenic than the *Aspergillus*-derived urate oxidase. Conjugation of human serum albumin (HSA) to therapeutic proteins has become a promising strategy to prolong circulation time in vivo. To develop a thermostable and long-circulating urate oxidase, we investigated the site-specific conjugation of HSA to AgUox based on site-specific incorporation of a clickable non-natural amino acid (frTet) and an inverse electron demand Diels–Alder reaction. We selected 14 sites for frTet incorporation using the ROSETTA design, a computational stability prediction program, among which AgUox containing frTet at position 196 (Ag12) exhibited enzymatic activity and thermostability comparable to those of wild-type AgUox. Furthermore, Ag12 exhibited a high HSA conjugation yield without compromising the enzymatic activity, generating well-defined HSA-conjugated AgUox (Ag12-HSA). In mice, the serum half-life of Ag12-HSA was approximately 29 h, which was roughly 17-fold longer than that of wild-type AgUox. Altogether, this novel formulated AgUox may hold enhanced therapeutic efficacy for several diseases.

## 1. Introduction

A high level of uric acid followed by its crystallization is related to tumor lysis syndrome, hyperuricemia, and gout [1,2,3]. Gout is a common type of inflammatory arthritis in adults, resulting from the formation of uric acid crystals in the joints and other tissues [1,2,3]. Therefore, the treatment of gout has focused on reducing serum uric acid levels, which has been effectively achieved by the injection of urate oxidase [3,4]. Urate oxidase (Uox, Enzyme Commission number: 1.7.3.3) is a peroxisomal liver enzyme that catalyzes the conversion of insoluble uric acid (0.06 g/L) to the more water-soluble 5-hydroxyisourate (10.6 g/L; predicted using ALOGPS) [5,6,7,8]. In humans, intravenous administration of Uox has been used for enzymatic therapy of hyperuricemia, supplementing the enzyme activity lost during hominoid evolution [3]. Rasburicase [9,10] and pegloticase [11,12,13] have been approved for the treatment of tumor lysis syndrome and gout, respectively. Rasburicase is a recombinant version of Uox derived from *Aspergillus flavus* that was demonstrated to be therapeutically superior to allopurinol for the control of uric acid levels in adult patients [9,10,11]. Pegloticase (marketed under the name Krystexxa) is a PEG-conjugated chimeric porcine–baboon Uox, with an extended serum half-life in vivo [12,13,14]. However, several concerns have been raised regarding PEG-conjugated therapeutics, such as the potential immunogenicity and toxicity of accumulated PEG molecules [15]. Human serum albumin (HSA) has low to no immunogenicity and is biodegradable. Furthermore, HSA has an exceptionally long serum half-life in humans (>3 weeks) via neonatal Fc receptor (FcRn)-mediated recycling [16,17,18,19,20]. Therefore, in order to overcome the potential issues of PEG conjugation, we previously reported that direct conjugation or indirect binding of HSA to Uox isolated from *A. flavus* (AfUox) resulted in a prolonged circulation time in vivo [21,22], enhancing its potential use as a therapeutic agent for gout. Direct HSA conjugation leads to a greater extension of circulation time than that achieved with indirect HSA binding via fatty acid conjugation [21,22]. However, the clinical applications of HSA-conjugated AfUox may be limited by its intrinsic immunogenicity and low thermostability [23]. Recently, Uox derived from *Arthrobacter globiformis* (AgUox) was determined to hold desirable properties for therapeutic development, including soluble expression in *Escherichia coli*, good solubility at neutral pH, low immunogenicity, and good thermostability [4,24]. We confirmed that wild-type AgUox is more thermostable than wild-type AfUox (Figure S1). To develop HSA-conjugated Uox with promising potential for clinical applications, we investigated site-specific HSA conjugation to AgUox. We hypothesized that the conjugation of HSA to a permissive site of AgUox would lead to high thermostability, low immunogenicity, prolonged circulation time in vivo (particularly in mice), and retained enzymatic activity. It was reported that HSA interacts with mouse FcRn, resulting in the long serum half-life in mice [25]. Furthermore, the attachment of HSA to insulin and glucagon-like peptide 1 extended the circulation time in mice, likely due to the HSA interactions with mouse FcRn [26,27].

## 2. Materials and Methods

### 2.1. Materials

Bactotryptone and yeast extract were obtained from BD Biosciences (San Jose, CA, USA). Ni-nitrilotriacetic acid (NTA) agarose was obtained from Qiagen (Hilden, Germany), and frTet (4-(1,2,3,4-tetrazin-3-yl) phenylalanine) was purchased from Aldlab Chemicals (Woburn, MA, USA). TCO–Cy3 was purchased from AAT Bioquest (Sunnyvale, CA, USA). Axially substituted trans-cyclooctene maleimide (TCO-maleimide, A) was purchased from FutureChem (Seoul, Korea). Disposable PD-10 desalting columns, HiTrap Q HP anion exchange columns, and Superdex 200 10/300 GL Increase size exclusion columns were purchased from Cytiva (Uppsala, Sweden). All other chemical reagents were purchased from Sigma-Aldrich (St. Louis, MO, USA), unless otherwise indicated.

### 2.2. Computational Analysis of the frTet Incorporation Site in AgUox

Screening of the frTet incorporation site was performed using the molecular modeling software PyRosetta (Python-based Rosetta molecular modeling package, Pyrosetta4, the PyRosetta Team at Johns Hopkins University, Baltimore, MD, USA) [28,29], which performed point mutation and energy scoring functions based on the AgUox structure (PDB ID: 2YZE). The amino acid sequence in wild-type (WT) AgUox was replaced with the Y (tyrosine) or W (tryptophan) sequence, and then the energy of the full atoms in the protein was calculated. The energy function in PyRosetta is based on Anfinsen’s hypothesis that native-like protein conformations represent a unique, low-energy, thermodynamically stable conformation. The score value represents the sum of the van der Waals force, attractive, repulsive energy, Gaussian exclusion implicit solvation, and hydrogen bonds (short, long range, backbone-side chain, and side chain) between atoms on different residues separated by distance.

### 2.3. Construction of Plasmids for Expression of AgUox-WT and AgUox-frTet Variants

AgUox was synthesized by Macrogen (Seoul, Korea) and cloned into pBAD for site-specific frTet incorporation, generating the pBAD_AgUox plasmid. To replace the site selected by PyRosetta scoring with amber codons, the site-directed mutagenesis polymerase chain reaction (PCR) was performed using the pBAD_AgUox vector as template. The primer pairs used are shown in Table S1.

### 2.4. Expression and Purification of AgUox-WT and AgUox-frTet Variants

To site-specifically incorporate frTet into AgUox, each mutant plasmid was transformed into C321ΔA.exp (pDule C11RS)-competent cells [30], generating C321ΔAexp (pDule C11RS) (pBAD_AgUox_Amb variants) *E. coli* cells. Transformants were cultured at 37 °C overnight in Luria broth medium containing ampicillin (100 μg/mL) and tetracycline (10 μg/mL). Pre-cultured *E. coli* cells were inoculated into identical fresh media. To induce protein expression, final concentrations of 1 mM and 0.4% of frTet and arabinose, respectively, were added to the medium, which reached an optical density of 0.5% (at 600 nm). The culture medium was incubated at 37 °C for 5 h with shaking, before being harvested via centrifugation at 5000 rpm for 10 min at 4 °C. AgUox-containing frTet variants were purified by immobilized metal affinity chromatography, using the interaction between Ni-NTA and His-tag, according to the manufacturer’s protocols. The expression and purification of AgUox-WT was performed similarly to that of the AgUox-frTet variants, without the addition of tetracycline and frTet.

### 2.5. Matrix-Assisted Laser Desorption Ionization Time-of-Flight Mass Spectrometry (MALDI-TOF MS) and Dye Labeling Analysis of AgUox Variants

Purified AgUox-WT and AgUox-frTet variants (0.4 mg/mL) were digested with trypsin. The trypsin-digested mixture was desalted using ZipTip C18 (Millipore, Billerica, MA, USA). The desalted trypsin-digested protein sample was mixed with 2,5-dihydroxybenzoic acid (DHB) solution (20 mg/mL of DHB in 3:7, (*v*/*v*) acetonitrile: 0.1% trifluoroacetic acid in water) in a 1:1 ratio. Then, 0.5 μL of this mixture was loaded onto a ground steel target (Bruker Corporation, Billerica, MA, USA) and molecular weight analysis was performed by MALDI-TOF MS (Bruker Corporation, Billerica, MA, USA).

To identify the IEDDA reactivity of the AgUox-frTet variants, purified AgUox-WT and AgUox-frTet variants were desalted with phosphate-buffered saline (PBS, pH 7.4) and then mixed with TCO-Cy3 at a molar ratio of 1:2 for 2 h at room temperature. Afterwards, the mixture, with or without the addition of TCO-Cy3, was subjected to sodium dodecyl sulfate polyacrylamide gel electrophoresis (SDS-PAGE). The gel underwent fluorescence analysis (excitation: 302 nm, filter 510/610 nm) in a ChemiDoc XRS+ System (Bio-Rad Laboratories, Hercules, CA, USA), followed by visualization after Coomassie brilliant blue (CBB) staining.

### 2.6. Enzymatic Activity Assay and Thermostability Assessment of AgUox-WT and AgUox-frTet Variants

AgUox variants (100 μL at 120 nM in enzyme activity assay buffer (50 mM sodium borate with 150 mM NaCl)) were mixed with 100 μL of 200 μM uric acid in enzyme activity assay buffer. The degradation of uric acid was then measured using the absorbance of the mixture solution at 293 nm. Enzyme activities were expressed as specific activity (U/mg AgUox). One unit (U) of activity was defined as the amount of enzyme that catalyzed the oxidation of 1.0 μmol of uric acid per minute at 25 °C. The serum activity of the AgUox-WT and AgUox-frTet variants was measured by an enzymatic activity assay of diluted serum in the enzyme assay buffer containing uric acid. Briefly, 10 μL of serum separated from whole blood at different time points was diluted in 90 μL of enzyme activity assay buffer and then mixed with 100 µL of 200 µM uric acid solution, and absorbance was measured at 293 nm. To measure the thermostability of AgUox variants, each variant was incubated for 10 days in PBS (pH 7.4) and subjected to the enzyme activity assay described above at 0, 5, and 10 days.

### 2.7. Generation of HSA-Conjugated AgUox-frTet Variants

HSA was subjected to the elimination of high-molecular weight aggregates using anion exchange chromatography (Hitrap Q HP column) in 20 mM Tris buffer (pH 7.0), as previously reported [21,31]. Purified HSA was desalted with PBS (pH 7.0), and reacted with TCO-MAL heterobifunctional crosslinker at a molar ratio of 1:4 for 2 h at room temperature. Afterwards, the mixtures were desalted with PBS (pH 7.4), generating the HSA-TCO conjugate. Purified AgUox-frTet variants were mixed with HSA-TCO at a molar ratio of 1:4 for 5 h at room temperature, and then analyzed by SDS-PAGE to identify the site-specific albumin conjugation yield. For further activity and pharmacokinetic studies, the HSA-conjugated AgUox-196frTet (AgUox-196HSA) was separated from the reaction mixture using size-exclusion chromatography. The elution peak corresponding to the Uox-HSA conjugate was used for an enzyme activity assay and pharmacokinetic studies after measuring the molecular weight by SDS-PAGE analysis.

### 2.8. Pharmacokinetic Studies

Briefly, 4.4 nmol (monomeric AgUox basis) of AgUox-WT or AgUox-HSA4 in 200 μL PBS (pH 7.4) was intravenously injected into the tail of young female BALB/c mice (*n* = 4). To evaluate the serum half-life of AgUox variants in vivo, retro-orbital blood collection was performed at 15 min and 3, 6, and 12 h for AgUox-WT; and 15 min and 3, 6, 12, 24, 48, and 72 h post-injection for AgUox-HSA. Serum activity was measured in serum isolated from the different whole blood samples collected.

## 3. Results and Discussion

### 3.1. Preparation of AgUox-WT and AgUox Containing frTet (AgUox-frTet) Variants

**Figure 1:**
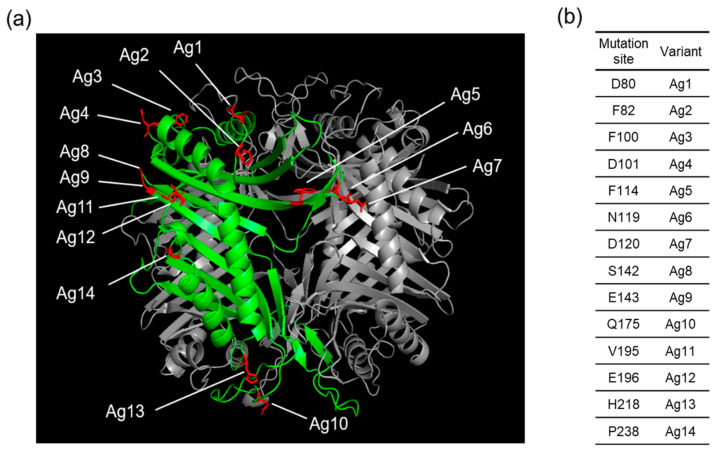
(**a**) Crystal structure of AgUox (PDB code: 2YZE) showing the selected sites for frTet incorporation. (**b**) The frTet incorporation sites and corresponding AgUox variants containing frTet are indicated in the table.

**Figure 2:**
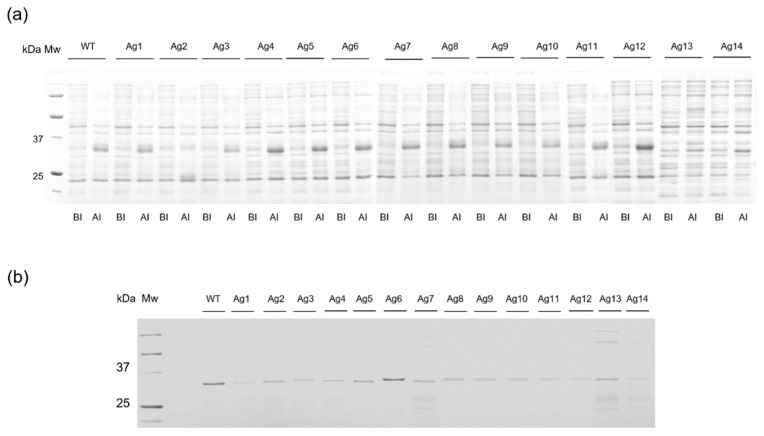
Expression and purification of AgUox-frTet variants. (**a**) Coomassie blue-stained protein gels of AgUox-WT and AgUox-frTet (Ag1–14) variants. Lanes: MW, molecular weight marker; BI, before induction; AI, after induction. (**b**) Image of Coomassie blue-stained protein gels of AgUox-WT and AgUox-frTet variants after purification.

As the first step for preparing HSA-conjugated AgUox variants, the optimal sites of AgUox for HSA conjugation were determined. In order to investigate the similarities between AfUox and AgUox, we performed amino acid sequence alignment and overlapped the crystal structures of AfUox and AgUox. The identity of the two amino acid sequences was only 38.5% (Figure S2). Due to the low identity, the crystal structures of AfUox and AgUox were poorly overlapped (Figure S3). Therefore, it was not straightforward to choose a site for frTet incorporation by comparing the amino acid sequence and crystal structures of the two Uox molecules. In the case of AfUox, the solvent accessibility and hydrophobicity of site were taken into consideration. However, a mutation often leads to misfolding or unfolding of a protein. Therefore, we performed a more systemic approach. Using PyRosetta, we calculated the energy score of AgUox variants containing a single mutation. Thus, the energy score of each variant was translated into its relative folding stability [29,32,33]. In order to mimic the mutation to frTet (a phenylalanine analog), the mutation to either Y or W was introduced to various sites of AgUox-WT. The top 14 sites for which the energy scores upon the mutation to both Y and W were greater than or comparable to that of AgUox-WT (Table S2), along with the 14 AgUox mutants containing frTet (AgUox-frTet) (named as Ag1–14, Figure 1), were identified. In order to prepare 14 AgUox-frTet variants, an amber codon was introduced to each of the14 sites of AgUox-WT by PCR-mediated mutagenesis. Then, C321delAexp *E. coli* cells [34] were co-transformed into pDule C11RS plasmid [35] encoding the engineered MjtRNA^Tyr^/MjTyrRS specific for ftTet as well as the vectors with each AgUox variant. The transformants were cultured to express each AgUox-frTet variant as described in ‘Materials and Methods’ (Section 2.3). In the CBB-stained protein gel, a molecular weight of 34 kDa, which corresponded to the monomeric AgUox, was detected in lanes of the cell lysate after induction and purified AgUox-frTet (Figure 2). Overall, these results demonstrate the successful expression and purification of AgUox-WT and AgUox-frTet variants.

### 3.2. Enzymatic Activity and Thermostability Assays of AgUox Variants

**Figure 3:**
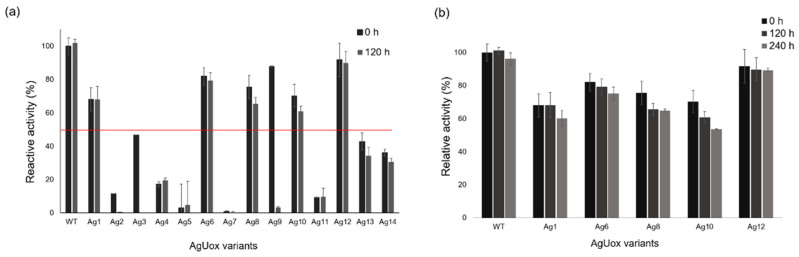
Thermostability assessment of AgUox-WT and AgUox-frTet variants. (**a**) Relative enzyme activity of AgUox-WT and AgUox-frTet (Ag1–14) variants in PBS monitored at 0 and 120 h. Red line indicates the 50% enzymatic activity of AgUox-WT. (**b**) Relative enzymatic activity of AgUox-WT and five AgUox-frTet variants (Ag1, 6, 8, 10, and 12) monitored at 0, 120, and 240 h. The relative activity of AgUox-frTet variants was normalized against the enzymatic activity of AgUox-WT.

To investigate whether the site-specific incorporation of frTet into AgUox affected its biological function, the enzymatic activities of purified AgUox-WT and AgUox-frTet variants were compared. The enzymatic activities of the AgUox-frTet variants varied between 1 and 93% relative to that of AgUox-WT (Figure 3a), indicating that the frTet incorporation site significantly affects the function of AgUox. The AgUox-frTet variants Ag1, 6, 8, 10, and 12 exhibited relatively high enzymatic activity (Figure 3a). The active sites of AgUox are located at the interfaces between monomers [36]. Noteworthily, the frTet incorporation sites of those variants (Ag1, 6, 8, 10, and 12) were far away from the active sites and interfaces between monomers.

AgUox-WT was reported to be thermostable [4]. In order to evaluate the thermostability of AgUox-frTet variants, the AgUox-frTet variants as well as AgUox-WT were incubated at 37 °C for five days, after which enzymatic activity assays were performed. As expected, no activity loss of AgUox-WT was observed after the 5-day incubation (Figure 3a). Among the 14 AgUox-frTet variants, Ag1, 6, 8, 10, and 12 exhibited more than 50% activity of AgUox-WT after the same period (Figure 3a). Those five variants were incubated for up to 10 days at 37 °C, after which Ag1, 6, 8, and 10 still showed an activity higher than 50% of AgUox-WT (Figure 3b). Although some activity loss was observed after the 10-day incubation, the Ag12 variant maintained an enzymatic activity similar to that of AgUox-WT (Figure 3b).

### 3.3. Confirmation of the Site-Specific frTet Incorporation to AgUox

**Figure 4:**
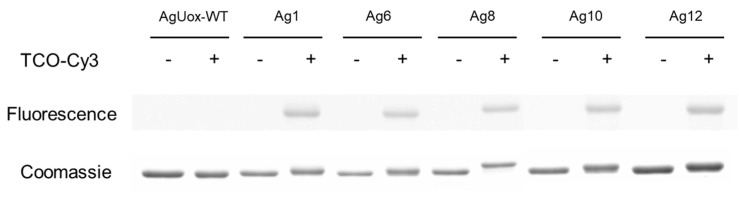
Incorporation of frTet into AgUox. Fluorescence (illumination λex = 302 nm, with wavelengths at 510 and 610 nm in Chemidoc XRS+ system) and Coomassie blue-stained protein gel for reaction mixture of TCO-Cy3 with AgUox-WT or AgUox-frTet (Ag) variants.

To confirm the frTet incorporation into each site on AgUox, we performed the fluorescence dye labeling of intact AgUox-frTet variants. As representative cases, the five AgUox-frTet variants with a relatively high activity (Ag1, 6, 8, 10, and 12) were analyzed. First, we performed the fluorescence dye labeling using TCO-Cy3 to confirm the IEDDA reactivity of AgUox-frTet variants. Evaluation of the fluorescent image of the protein gel revealed no band in AgUox-WT samples, indicating no IEDDA reactivity of AgUox-WT (Figure 4). In contrast, the Ag1, 6, 8, 10, and 12 variants clearly exhibited the band in both the fluorescence image of protein gel and the CBB-stained protein gel, confirming the IEDDA reactivity of AgUox-frTet variants (Figure 4).

Next, frTet incorporation was further confirmed by MALDI-TOF MS of trypsin-digested AgUox-frTet (Ag1, 6, 8, 10, and 12) variants using AgUox-WT as control (Figure S4). In the mass spectra of trypsin-digested AgUox-frTet variants, the observed masses of fragments containing frTet matched well with the respective theoretical values with a deviation of less than 0.05% (Table S3). These results confirm the site-specific incorporation of frTet into specific sites of AgUox-frTet variants.

### 3.4. Site-Specific HSA-Conjugation to AgUox-frTet

**Figure 5:**
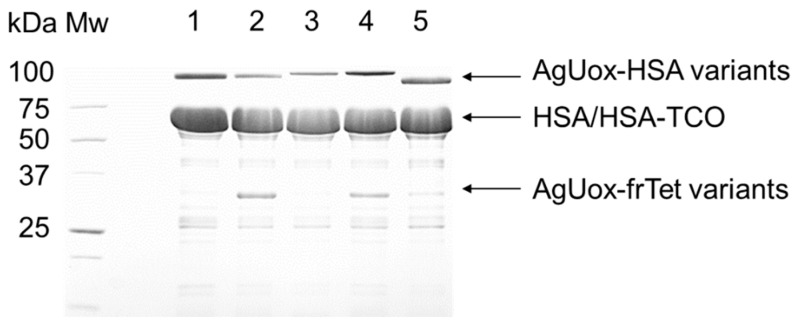
SDS-PAGE analysis of AgUox-HSA conjugate variants. The protein gel was visualized using Coomassie blue staining. Lanes: MW, molecular weight marker; 1, AgUox-HSA from Ag1 variant; 2, AgUox-HSA from Ag6 variant; 3, AgUox-HSA from Ag8 variant; 4, AgUox-HSA from Ag10 variant; 5, AgUox-HSA from Ag12 variant.

To prepare HSA-conjugated AgUox, we used the heterobifunctional crosslinker, TCO-MAL. First, TCO-MAL was conjugated to the free cysteine at position 34 (Cys34) of HSA via Michael addition. Since the only free cysteine (Cys34) on the HSA surface is located away from the FcRn binding domain, it has been frequently used for bioconjugation [37,38,39,40]. Then, TCO-HSA was conjugated to the purified AgUox-frTet variants via the IEDDA reaction to generate AgUox-HSA conjugates. The reaction mixtures were subjected to SDS-PAGE analysis (Figure 5). In the CBB-stained protein gel, the bands for HSA-conjugated AgUox-frTet (Ag1, 6, 8, 10, and 12) variants were clearly observed in the range of 100–120 kDa (Figure 5). In case of Ag1, 8, and 12 variants, no band of monomeric AgUox was observed, indicating the almost complete conjugation of AgUox to HSA. In the case of Ag6 and 10, the band of AgUox monomer was observed within 25–37 kDa, indicating poor AgUox conjugation to HSA. The trend in the HSA conjugation yield of Ag variants, except for Ag8, was similar to that of solvent accessibility (Ag1, 6, 8, 10, and 12: 0.93, 0.51, 0.9, 0.85, and 0.92, respectively). Since the Ag12 variant exhibited the highest HSA conjugation yield, as well as the highest enzymatic activity, it was selected for further characterization. To confirm the generation of Ag12-HSA, we performed MALDI-TOF MS analysis of the reaction mixture generating Ag12-HSA as well as AgUox-WT. The observed mass of intact AgUox-WT in the mass spectrum was 33,312 Da, which is quite consistent with its expected mass (33,305 Da) with a deviation of 0.03% (Figure S5a). In the mass spectrum of the conjugation mixture of Ag-HSA, three bands were observed. The band at 66,779 Da was expected to be that for HSA-TCO, as it matched well with its theoretical mass of 66,770 Da. The observed masses of Ag12 and Ag-HSA were 33,394, 66,779, and 100,378 Da, which are quite consistent with their expected masses (33,403, 66,770, and 100,363 Da), respectively (Figure S5b).

### 3.5. Enzymatic Activity of the AgUox-HSA Conjugate

**Figure 6:**
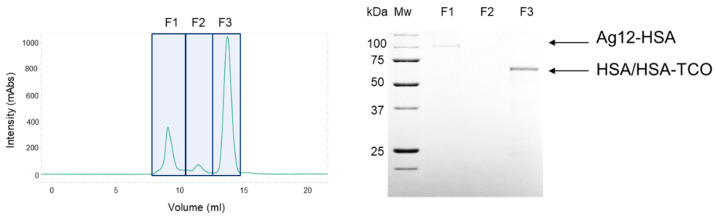
Purification of HSA-conjugated Ag12 variant (Ag12-HSA). Size-exclusion chromatography of Ag12-HSA conjugate mixture (**right**) and SDS-PAGE analysis of the eluted fractions (F1–F3) of Ag12-HSA (**left**). The protein gel was stained by Coomassie blue for the visualization of protein bands. Lanes: MW, molecular weight marker.

**Figure 7:**
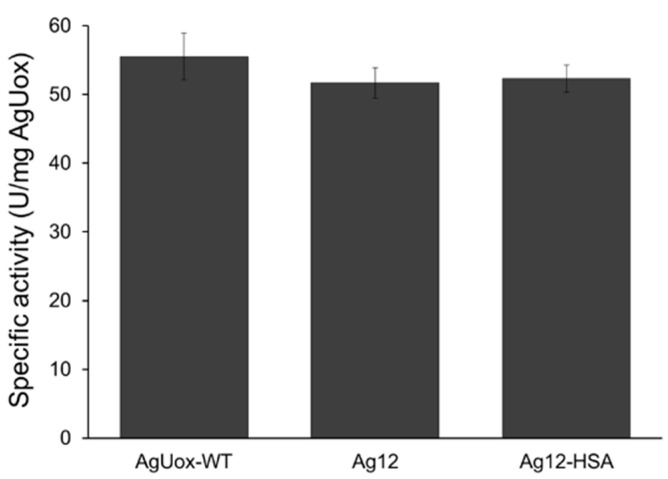
Specific activity of AgUox-WT, Ag12, and Ag12-HSA. Experiments were performed in quadruplicate, and error bars indicate the standard deviation.

We purified the HSA-conjugated Ag12 variant (Ag12-HSA) from the reaction mixture using size-exclusion chromatography. The eluted fractions in the chromatograms were analyzed by SDS-PAGE (Figure 6). The two major peaks indicate the Ag12-HSA and unreacted HSA-TCO, respectively, whereas the peak for Ag12 monomer was not detected, indicating that the HSA conjugation yield was high. The specific activities of Ag12 and Ag12-HSA were 51.7 and 52.3 U/mg AgUox, respectively, which were approximately 93% of that of AgUox-WT (Figure 7). The specific activity of AgUox variants was calculated based on the weight of AgUox in order to avoid the underestimation of the specific activity of AgUox-HSA conjugates due to the weight of HSA molecules. These results indicate that the Ag12 variant is suitable for site-specific HSA conjugation with the retained enzymatic activity.

### 3.6. Pharmacokinetic Study of AgUox-WT and Ag12-HSA

**Figure 8:**
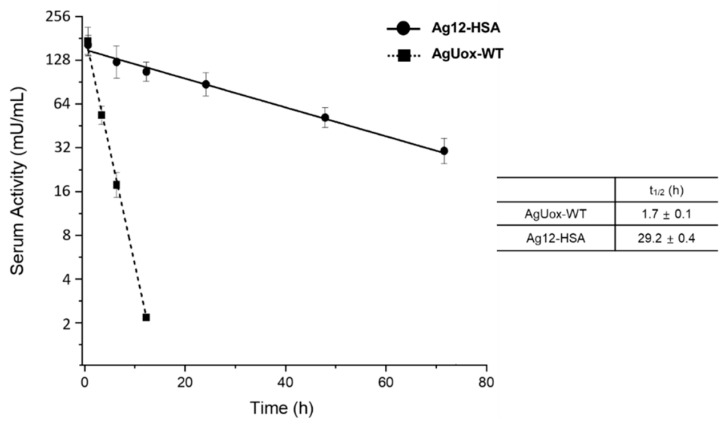
Pharmacokinetic studies of AgUox-WT and Ag12-HSA in mice. Enzymatic activity of residual AgUox-WT and Ag12-HSA conjugate samples was measured at 15 min; at 3, 6, and 12 h for AgUox-WT; and 15 min and 12, 24, 48, and 72 h for Ag12-HSA. The samples for pharmacokinetic studies were intravenously injected into BALB/c female mice (*n* = 4). Error bars indicate the standard deviation.

We measured the serum half-lives of AgUox-WT and Ag12-HSA after intravenous administration to mice. Moreover, the enzymatic activity of AgUox species in the serum samples was monitored. The serum half-life of AgUox-WT was about 1.7 h (Figure 8), which was longer than that of AfUox-WT (1.3 h) [30]. We also observed that the serum half-life of the Ag12-HSA conjugate was 29 h, which was approximately 17-times higher than that of AgUox-WT (Figure 8), indicating that HSA conjugation effectively prolonged the serum half-life of AgUox. Noteworthily, the serum half-life of Ag12-HSA conjugate was longer than that of AfUox-HSA (21 h) [30]. Considering that both Ag12-HSA and AfUox-HSA have four HSA molecules conjugated to each Uox molecule with the same linker, we believe that the difference observed in their serum half-life results from thermostability differences. In the case of AfUox-HSA, the serum half-life of Ag12-HSA was assessed by measuring the enzyme activity of the AgUox variant remaining in the serum. Taken together, these results highlight the thermostability of AgUox and how it retains its enzymatic activity in vivo. Furthermore, these data indicate that the conjugation of HSA to AgUox, which has high thermostability, results in a significantly longer serum half-life in vivo.

## 4. Conclusions

AgUox is a promising therapeutic candidate for gout treatment because of its high thermostability and low immunogenicity. To further develop AgUox as a therapeutic agent, we achieved site-specific HSA conjugation to AgUox, resulting in the significantly prolonged circulation time in vivo compared with AgUox-WT and AfUox-HSA, likely due to the high thermostability of AgUox and the FcRn-mediated recycling of HSA. We demonstrated that the computational stability prediction of AgUox variants containing frTet successfully led to identification of 14 stable AgUox-frTet variants. As expected, approximately half of these variants retained enzymatic activity and relatively high thermostability. In particular, AgUox-196frTet (Ag12) showed enzyme activity and thermostability comparable to those of AgUox-WT. Pharmacokinetic studies further showed that the serum half-life of Ag12-HSA was extended to 29 h, which was approximately 17 times longer than that of AgUox-WT. Hence, we believe that the HSA-conjugated AgUox would be a good therapeutic candidate for severe gout treatment. Since the Uox-based therapeutics are very expensive compared to other small molecule-based urate lowering drugs, their use would be limited to patients with severe and refractory gout.

## Data Availability

The supporting data presented in this study are available in Supplementary Materials.

## Supplementary Materials

The following are available online at https://www.mdpi.com/article/10.3390/pharmaceutics13081298/s1, Figure S1. Time-course enzymatic activity of AfUox-WT and AgUox-WT; Figure S2. Alignment of the amino acid sequences of AgUox and AfUox; Figure S3. The crystal structures of (**a**) AgUox, (**b**) AfUox, and (**c**) The overlapped structures of AgUox and AfUox; Figure S4. Matrix-assisted laser desorption/ionization-time of flight mass spectra (MALDI-TOF MS) of trypsin-digested (**a**) Ag1, (**b**) Ag6, (**c**) Ag8, (**d**) Ag10, and (**e**) Ag12; Figure S5. MALDI-TOF mass spectra of AgUox-WT (**a**) and the conjugate mixture generating Ag12-HSA (**b**); Table S1. Primers used for the site-directed mutagenesis of AgUox; Table S2. Rosetta scores of AgUox after point mutation into tryptophan or tyrosine; Table S3. List of masses of trypsin-digested AgUox-frTet variants.
