# Supplementary material for: Thermostable and Long-Circulating Albumin-Conjugated Arthrobacter globiformis Urate Oxidase
Source: Pharmaceutics. 2021 Aug 19;13(8):1298. doi: 10.3390/pharmaceutics13081298 (PMC8400835; doi:10.3390/pharmaceutics13081298)
Supplement: Supplementary file 1 [file pharmaceutics-13-01298-s001.zip › pharmaceutics-1278126-supplementary.pdf]

pharmaceutics

MDPI

Supplementary Materials: Thermostable and long-circulating albumin-conjugated Arthrobacter globiformis urate oxidase

Byungseop Yang and Inchan Kwon

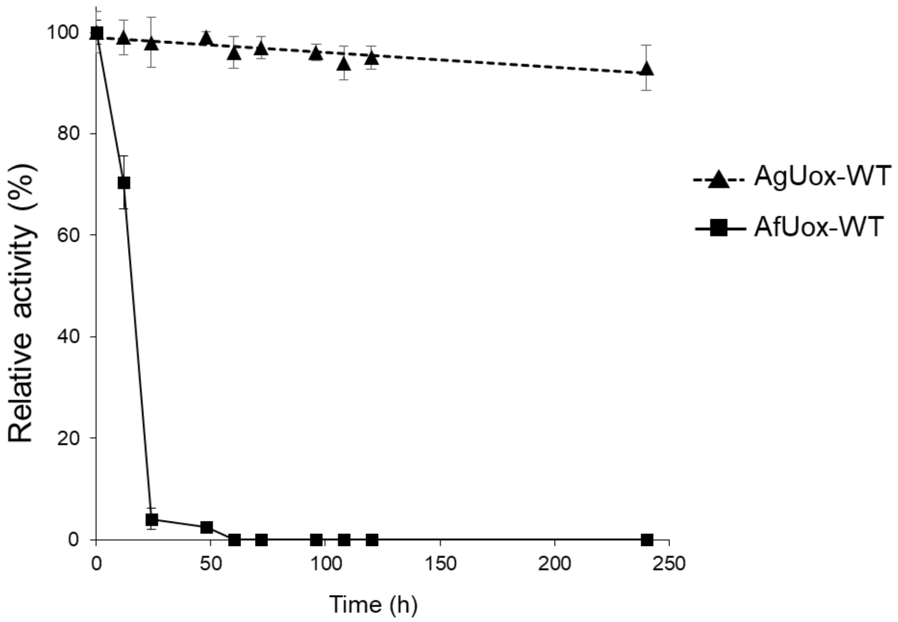

Figure S1. Time-course enzymatic activity of AfUox-WT and AgUox-WT. The enzymatic activity was determined using enzymatic activity assay, as described in 'Material and Methods' (section 2.6). Relative activity of AfUox-WT and AgUox-WT was normalized to that of AfUox-WT.

```
AgUox   MTATAETSTGTKVVLGQNQYGKAEVRLVKVTRNT--ARHEIQDLNVTSQLRGDFEAAHTA   58
AfUox   -----------MSAVKAARYGKDNVRVYKVHKDEKTGVQTVYEMTVCVLLEGEIETSYTK   49
                     .:   :*** :**: ** ::   . : : ::.*   *.*::*:::*

AgUox   GDNAHVVATDTQKNTVYAFARDGFAT-TEEFLLRLGKHFTEGFDWVTGGRWAAQQFFWDR   117
AfUox   ADNSVIVATDSIKNTIYITAKQNPVTPPELFGSILGTHFIEKYNHIHAAHVNIVCHRWTR   109
        .**: :****: ***:*  *::. .*  * *   **.** * :: : ..:     . * *

AgUox   IN----DHDHAFSRNKSEVRTAVL-EISGSEQAIVAGIEGLTVLKSTGSEFHGFPRDKYT   172
AfUox   MDIDGKPHPHSFIRDSEEKRNVQVDVVEGKGIDIKSSLSGLTVLKSTNSQFWGFLRDEYT   169
        ::     * *:* *:..* *.. :  :.*.   * :.:.********.*:* ** **:**

AgUox   TLQETTDRILATDVSARWRYNTV---------EVDFDAVYASVRGLLLKAFAETHSLALQ   223
AfUox   TLKETWDRILSTDVDATWQWKNFSGLQEVRSHVPKFDATWATAREVTLKTFAEDNSASVQ   229
        **:** ****:***.* *:::..           .***.:*:.* : **:*** :* ::*

AgUox   QTMYEMGRAVIETHPEIDEIKMSLPNKHHFLVDLQPFG----QDNPNEVFYAADRPYGLI   279
AfUox   ATMYKMAEQILARQQLIETVEYSLPNKHYFEIDLSWHKGLQNTGKNAEVFAPQSDPNGLI   289
         ***:*.. ::  :  *: :: ******:* :**. .      .:  ***   . * ***

AgUox   EATIQREGSRADHHHHHH-                                           297
AfUox   KCTVGRSSLKSKLHHHHHH                                           308
        :.*: *.. ::. *****
```

(Identity: 38.5 %)

Figure S2. Alignment of the amino acid sequences of *Arthrobacter globiformis* urate oxidase (AgUox) and *Aspergillus flavus* urate oxidase (AfUox) using Clustal W [1]. The albumin conjugation sites in the AgUox and AfUox are marked in red and blue, respectively.

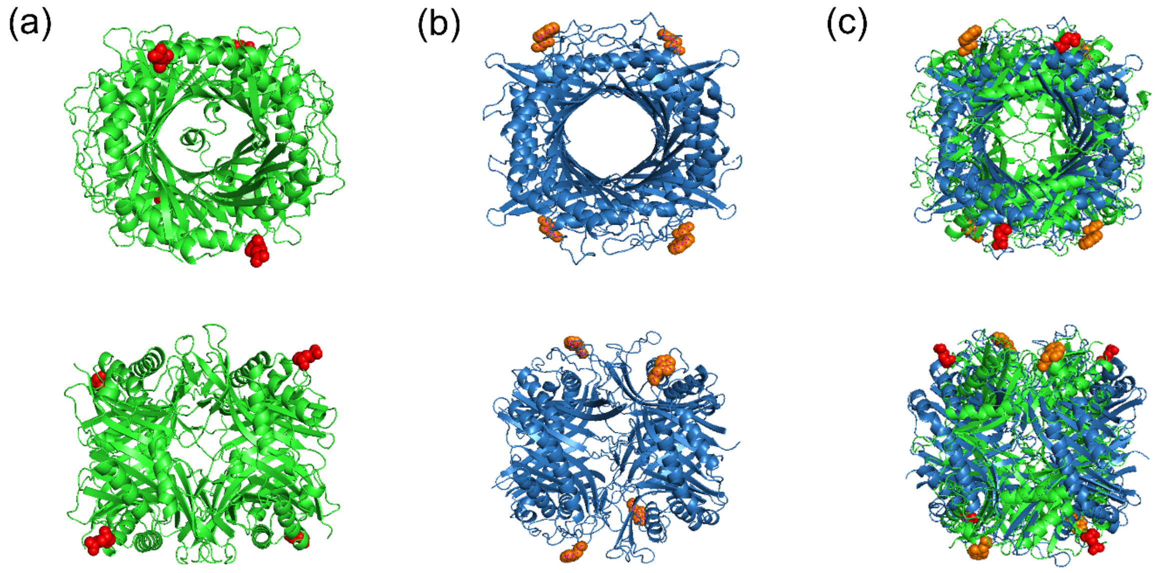

Figure S3. The crystal structures of urate oxidases from (a) Arthrobacter globiformis (AgUox) (PDB ID: 2YZE) and (b) Aspergillus flavus (AfUox) (PDB ID: 1WS2) (top panel: front view; bottom panel: side view). (c) The overlapped structures of AgUox and AfUox. The frTet incorporation sites are marked by spheres in red (E196 in AgUox) or orange (W174 in AfUox). The structures are visualized by PyMol [The PyMOL Molecular Graphics System, Version 1.2r3pre, Schrödinger, LLC].

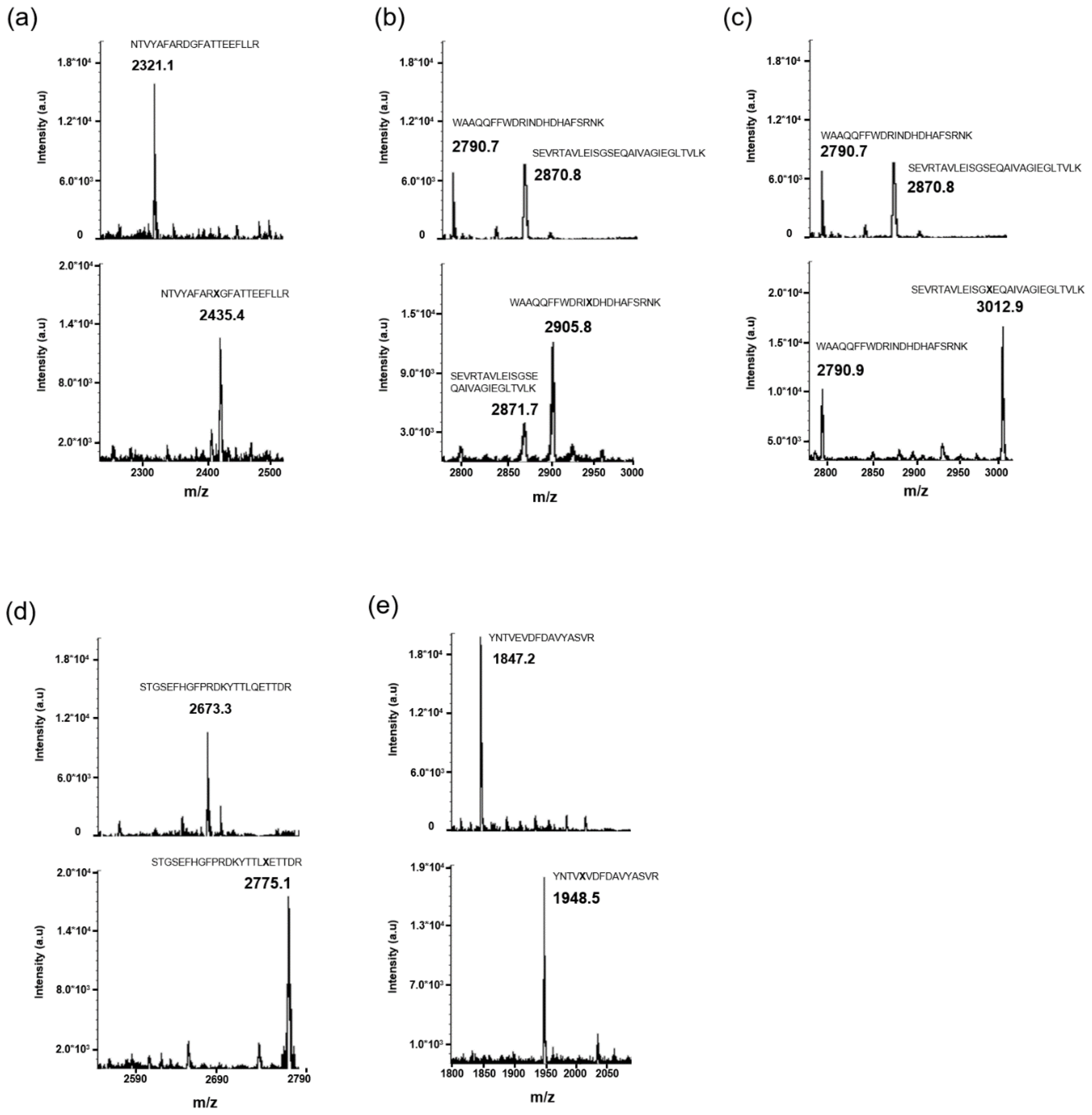

Figure S4. Matrix-assisted laser desorption/ionization-time of flight mass spectra (MALDI-TOF MS) of trypsin-digested (**a**) Ag1, (**b**) Ag6, (**c**) Ag8, (**d**) Ag10, and (**e**) Ag12. AgUox-WT was used as the control.

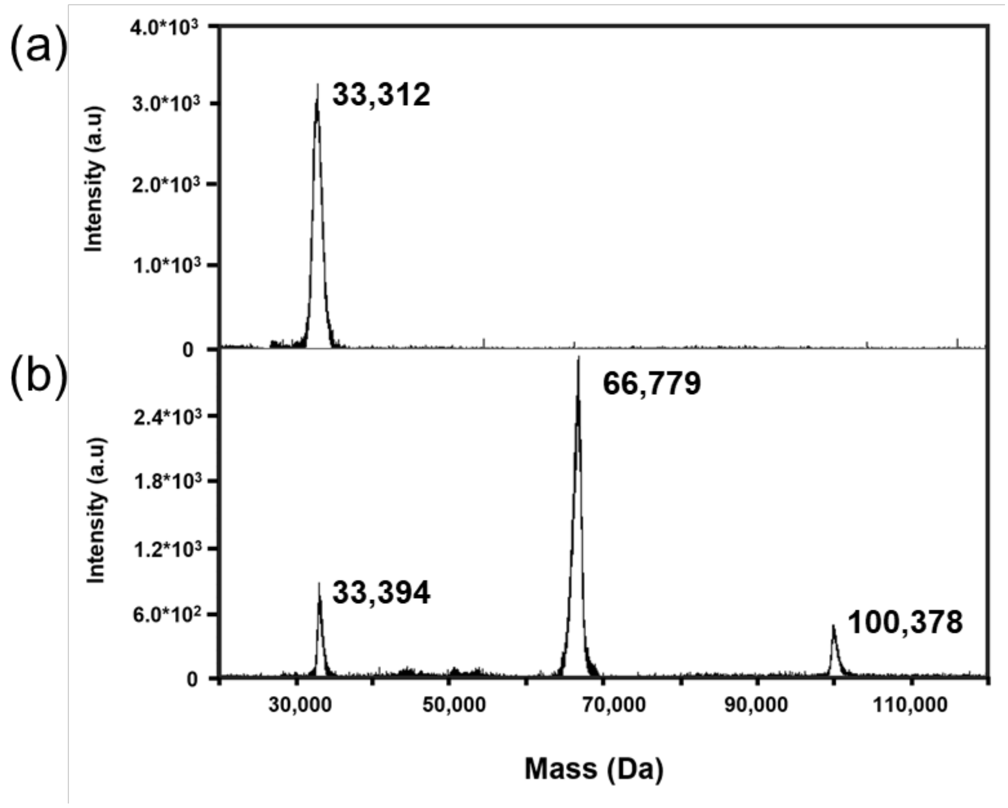

Figure S5. MALDI-TOF mass spectra of AgUox-WT (a) and the conjugate mixture generating Ag12-HSA (b). The samples were mixed with sinapinic acid (SA) matrix solution 2 (20 mg/mL of SA in 30:70 (v/v) acetonitrile: trifluoroacetic acid 0.1% in water) in a 1:1 ratio and then loaded onto ground steel target plate (Bruker Corporation, Billerica, MA, USA) coated with SA matrix solution 1 (20 mg/mL of SA in ethanol). The mass spectra of samples were obtained using a Microflex MALDI-TOF/MS (Bruker Corporation, Billerica, MA, USA).

Table S1. Primers used for the site-directed mutagenesis of AgUox

Variants	Forward	Reverse
AgUox_80Amb (Ag1)	5′–CGTGGCGAAACCCTAACGCGCGAACGCGTAC–3′	5′–GTACGCGTTCGCGCGTTAGGGTTTCGCCACG–3′
AgUox_82Amb (Ag2)	5′–TTCAGTCGTGGCCTAACCATCACGCGCGAACG–3′	5′–CGTTCGCGCGTGATGGTTAGGCCACGACTGAA–3′
AgUox_100Amb (Ag3)	5′–CCACCGGTAACCCAGTCCTAGCCTTCGGTAAAGTGT–3′	5′–ACACTTTACCGAAGGCTAGGACTGGGTTACCGGTGG–3′
AgUox_101Amb (Ag4)	5′–CACCGGTAACCCACTAAAAGCCTTCGGTAAAGTGTTTGCCC–3′	5′–GGGCAAACACTTTACCGAAGGCTTTTAGTGGGTTACCGGTG–3′
AgUox_114Amb (Ag5)	5′–TACGGTCCCACTAGAACTGCTGGGCAGCCCA–3′	5′–TGGGCTGCCCAGCAGTTCTAGTGGGACCGTA–3′
AgUox_119Amb (Ag6)	5′–ATGCATGGTCGTGGTCCTAGATACGGTCCCAGAAG–3′	5′–CTTCTGGGACCGTATCTAGGACCACGACCATGCAT–3′
AgUox_120Amb (Ag7)	5′–GAGAATGCATGGTCGTGCTAGTTGATACGGTCCCAGA–3′	5′–TCTGGGACCGTATCAACTAGCACGACCATGCATTCTC–3′
AgUox_142Amb (Ag8)	5′–CAGCTACGATCGCCTGTTCCTAACCAGAGATTTCCAGTAC–3′	5′–GTACTGGAAATCTCTGGTTAGGAACAGGCGATCGTAGCTG–3′
AgUox_143Amb (Ag9)	5′–GCCAGCTACGATCGCCTGCTAAGAACCAGAGATTTCCAG–3′	5′–CTGGAAATCTCTGGTTCTTAGCAGGCGATCGTAGCTGGC–3′
AgUox_175Amb (Ag10)	5′–ACGGTCGGTGGTTTCCTACAGCGTGGTATATTTAT–3′	5′–ATAAATATACCACGCTGTAGGAAACCACCGACCGT–3′
AgUox_195Amb (Ag11)	5′–CAGCGTCGAAGTCCACTTCCTAGGTGTTGTAACGCCAACGG–3′	5′–CCGTTGGCGTTACAACACCTAGGAAGTGGACTTCGACGCTG–3′
AgUox_196Amb (Ag12)	5′–GTCGAAGTCCACCTATACGGTGTTGTAACGCCAACGG–3′	5′–CCGTTGGCGTTACAACACCGTATAGGTGGACTTCGAC–3′
AgUox_218Amb (Ag13)	5′–CAGGGCCAGGGACTAAGTTTCTGCGAATGCTTTCAGCA–3′	5′–TGCTGAAAGCATTCGCAGAAACTTAGTCCCTGGCCCTG–3′
AgUox_238Amb (Ag14)	5′–CTTGATTTCGTCAATTTCCTAGTGGGTCTCGATAACCGCG–3′	5′–CGCGGTTATCGAGACCCACTAGGAAATTGACGAAATCAAG–3′

Table S2. ROSETTA scores of AgUox after point mutation into (**a**) tryptophan or (**b**) tyrosine

(a)

Mutation site	ROSETTA score
E196	2582.93
E143	2535.44
WT	2523.29
P238	2518.78
F114	2508.66
V195	2505.66
N119	2501.68
S142	2501.24
H218	2499.75
Q175	2498.62
D80	2490.78
D120	2486.32
F100	2479.7
D101	2479.7
F82	2408.17

(b)

Mutation site	ROSETTA score
E196	2596.28
E143	2547.95
N119	2526.71
WT	2523.29
F100	2517.66
D101	2517.16
F114	2514.91
F82	2511.90
Q175	2511.29
P238	2511.12
S142	2509.92
D80	2507.26
D120	2503.98
H218	2499.57
V195	2145.17

Table S3. List of masses of trypsin digested AgUox-frTet variants

Variants	Mutation site	Theoretical mass (m/z)	Observed mass (m/z)	sequence
Ag1	D80	2435.3	2435.4	NTVYAFARD_{80}GFATTEEFLLR
Ag6	N119	2904.4	2905.8	WAAQQFFWDRIN_{119}DHDHAFSRNK
Ag8	S142	3011.8	3012.9	SEVRTAVLEISGS_{142}EQAIVAGIEGLTVLK
Ag10	Q175	2774.4	2775.1	STGSEFHGFPRDKYTTLQ_{175}ETTDR
Ag12	E196	1948.0	1948.5	YNTVE_{196}VDFDAVYASVR

References

1. Madeira, F.; Park, Y. mi; Lee, J.; Buso, N.; Gur, T.; Madhusoodanan, N.; Basutkar, P.; Tivey, A.R.N.; Potter, S.C.; Finn, R.D.; et al. The EMBL-EBI search and sequence analysis tools APIs in 2019. *Nucleic Acids Res*. **2019**, 47, 636–641, doi:10.1093/NAR/GKZ268.
